# What are the priorities for improving cataract surgical outcomes in Africa? Results of a Delphi exercise

**DOI:** 10.1007/s10792-017-0599-y

**Published:** 2017-06-20

**Authors:** J. C. Buchan, W. H. Dean, A. Foster, M. J. Burton

**Affiliations:** International Centre for Eye Health, London School of Hygiene & Tropical Medicine, Keppel Street, London, WC1E 7HT UK

**Keywords:** Cataract, Delphi technique, Quality improvement, Sub-Saharan Africa

## Abstract

**Purpose:**

The quality of cataract surgery delivered in sub-Saharan Africa (SSA) is a significant constraint to achieving the elimination of avoidable blindness. No published reports from routine SSA cataract services attain the WHO benchmarks for visual outcomes; poor outcomes (<6/60) often comprise 20% in published case series. This Delphi exercise aimed to identify and prioritise potential interventions for improving the quality of cataract surgery in SSA to guide research and eye health programme development.

**Methods:**

An initial email open-question survey created a ranked list of priorities for improving quality of surgical services. A second-round face-to-face discussion facilitated at a Vision 2020 Research Mentorship Workshop in Tanzania created a refined list for repeated ranking.

**Results:**

Seventeen factors were agreed that might form target interventions to promote quality of cataract services. Improved training of surgeons was the top-ranked item, followed by utilisation of biometry, surgical equipment availability, effective monitoring of outcomes of cataract surgery by the surgeon, and well-trained support staff for the cataract pathway (including nurses seeing post-operative cases).

**Conclusion:**

Improving the quality of cataract surgery in SSA is a clinical, programmatic and public health priority. In the absence of other evidence, the collective expert opinion of those involved in ophthalmic services regarding the ranking of factors to promote quality improvement, refined through this Delphi exercise, provides us with candidate intervention areas to be evaluated.

## Introduction

One in three of the world’s 32.4 million blind people (<3/60 presenting visual acuity (VA) in the better-seeing eye) are blind due to cataract, and this proportion is closer to one half in sub-Saharan Africa (SSA) [1, 2]. In addition, there are many millions more who have significant visual impairment from cataract. This is despite an effective, low-cost cure for cataract having been known for decades.

The availability of cataract surgical services in SSA is by no means universal, but even where services are available, uptake has mostly been below the level required for elimination of cataract blindness [3]. Cataract surgical rates (CSR) of around 500 operations/million population/annum are frequently reported, well below the target of 2000 that has been suggested by the World Health Organisation (WHO) [4, 5]. A commonly cited barrier to acceptance of surgery is concern about poor outcomes from surgery amongst potential beneficiaries [6–9].

The WHO defines the quality of outcomes as “good” if the vision is 6/18 or better and “poor” if it is <6/60. WHO outcome quality targets have been set of >80% “good” uncorrected outcome and/or 90% “good” best-corrected acuity, >4 week post-operatively and a maximum of 5% having a “poor” outcome [10]. Although large case series have been published from higher volume settings, demonstrating 79% “good” uncorrected outcomes (by a single surgeon doing >1455 adult cataracts per year) [11] and 89% best-corrected (in a unit doing >1800 cataracts per year) [12], to our knowledge, there are no published series of cataract cases from routine African hospital services that attain the WHO benchmarks. Rates of “poor” outcomes typically sit over 20% (range 14.6–44% from a review of case series) [13]. This is not the experience of the rest of the world, with recent international studies showing that the proportion of poor outcomes is a particular problem for SSA [14, 15].

If 20% of operated cataract cases fail to attain 6/60, it can be expected that this will generate some negative publicity in the general population that might then discourage uptake of surgery in an unhelpful feedback loop. Improving the quality of surgery on offer in SSA is therefore critical to the goal of increasing the quantity of surgery being performed and the reduction in cataract blindness.

There is an established link between the volume of surgery performed by a surgeon and the outcomes of surgery; the largest published study reported an eightfold increase in the complication rate for the least active surgeons (50–250 cataracts per year) when compared to the most active (>1000 cataracts per year) [16].

In grading systems of the quality of evidence, “expert opinion” is considered the poorest quality source (www.cebm.net/oxford-centre-evidence-based-medicine-levels-evidence-march-2009). However, where the evidence base is weak it may be the only option or at least offer a starting point to those wishing to further develop the evidence base.

To improve upon an unrefined “expert opinion”, the Delphi method provides a structured communication methodology that utilises a group of experts to predict or prioritise variables in an iterative process [17]. Its use in health care and research is increasing, and in the ophthalmic literature, it has been used to select clinical indicators for evaluation of disease progression and to guide selection of outcome measures by international research communities [18–20].

Given cataract’s pre-eminence as a global cause of blindness, there is a relative paucity of studies exploring interventions to improve outcomes in low- and middle-income countries. The purpose of this study was to generate consensus, using a Delphi process, around what factors might have the strongest influence on the outcomes of surgery and therefore form appropriate focuses for intervention.

## Methods

The population of experts we sought to draw from were those with clinical, programme management and research experience within SSA. To provide a greater breadth of experience and background to give a broader range of perspectives, we also included experts with experience in ophthalmic service delivery development and surgical training in SSA, but who currently work in non-African health systems.

A Vision 2020 Research Mentorship Workshop had been arranged in Moshi, Tanzania, in January 2017. There were 20 delegates from several ophthalmology training centres in Tanzania, Kenya and Uganda, within the College of Ophthalmologists of East, Central and Southern Africa (14), International Centre for Eye Health at the London School of Hygiene & Tropical Medicine (5), UK National Health Service (1) and the Royal College of Ophthalmologists UK (1). This therefore provided a diverse and experienced group to participate in this exercise.

The first round of the process was undertaken through an email survey. Delegates were emailed in advance of the meeting by an external administrator.

The rationale was stated by explaining that “Many involved with providing cataract surgery in sub-Saharan Africa find that the WHO benchmarks for visual outcomes are difficult to attain. In order to identify interventions that will help improve outcomes, we would be grateful for your opinion regarding what areas most need attention.”

Delegates were posed an open question: “What changes can you suggest that would improve the quality of cataract surgery and the patient outcomes in your country? This is not specifically looking for changes you would like to see your own eye department, but for anyone currently offering cataract surgery in your country (the changes you suggest could relate to any part of the programme from the training of surgeons, management of hospitals or community-based services, equipment, the surgical techniques themselves or any aspect of clinical care before, during or after the surgery—anything you think will improve outcomes for patients)”.

Delegates were asked to rank their responses from the most important factor to the least important. The responses were emailed to an administrator, who removed identifying information, so that anonymity in response analysis was both perceived and achieved. Responses were collated by a researcher who was not taking part in the subsequent face-to-face round. No contributor’s response was therefore identifiable. The lists of factors received were then themed and similar responses coalesced and scored. From each candidate’s list, 10 points were allocated to the factor deemed most important, 9 points for the second placed factor, 8 points for the third and so on; no candidate offered more than 10 suggestions. Points were then totalled and a first-round ranking generated.

In the next stage of the Delphi process, the list was presented to the delegates for plenary discussion and refinement prior to the second iteration of grading. The discussion was facilitated in a face-to-face session to consider each factor in turn.

After the discussion, delegates were then asked to grade factors on the refined list from most important to least important. These ranked responses were then again collated, scored and analysed.

## Results

The first-round open-question email survey produced suggestions from 7 of the 18 people surveyed (response rate 39%). Two researchers involved in this study were excluded from the list of 20 delegates. The responses were synthesised into 18 proposed factors that, if developed, were perceived to “be contributory to the provision of good-quality cataract surgery”. The ranked factors are listed in Table 1.

**Table 1:** First-round scoring of factors perceived to be important to high-quality cataract services

Proposed factor to improve the quality of cataract surgery and patient outcomes	Cumulative score
Biometry	40
Well-trained surgeons	39
Equipment (non-consumable) such as cataract sets/microscopes	34
Effective monitoring of outcomes of cataract surgery by the surgeon	32
High volumes of patients (e.g. from outreach programs, community referral networks)	22
Consumables (e.g. viscoelastics, trypan blue)	21
Well-trained support staff for cataract pathway (including nurses seeing post-operative cases)	20
Refresher training available to surgeons (e.g. wet labs)	17
Post-operative refraction/monitoring of refractive outcomes	14
Availability of broad range of low-cost IOL	14
Opportunity for anonymous feedback from patients to their cataract surgeon	10
External (e.g. MOH) monitoring of cataract surgical outcomes	9
Fixed facilities (well-functional base operating theatre)	9
Vitrector	8
Proper case selection	7
Increase number of sub-specialised ophthalmologists (e.g. VR surgeons to deal with complications)	9
Close follow-up	5
Patient education	4

During the facilitated discussion, two factors (“biometry” and “availability of a broad range of low-cost intra-ocular lenses (IOL)”) were amalgamated as one without the other has little meaning. No factor was discarded outright, and no new factors were proposed at the meeting. Following discussion, the facilitator distributed lists of factors, and responses were obtained from 12 of the 14 delegates (response rate 86%). Allocation of points was again undertaken. The 12 delegates each independently ordered the factors from most important to least important. The most important was given a score of 17, incrementing 1 point less for each subsequent factor listed. A maximum score of 204 was therefore possible, as shown in Table 2. In each round of the process, the majority of respondents were African clinicians working currently in SSA, and the respondents not currently resident in SSA had each lived a minimum of 5 years in SSA and were all currently actively engaged in SSA ophthalmic research or clinical service development.

**Table 2:** Second-round scoring of factors perceived to be important to high-quality cataract services

Rank	Proposed factor to improve the quality of cataract surgery and patient outcomes	Score
1	Improved training of surgeons	196
2	Biometry	182
3	Equipment (non-consumable) (e.g. cataract instruments/microscopes)	173
4	Effective monitoring of outcomes of cataract surgery by the surgeon	158
5	Well-trained support staff for cataract pathway (including nurses seeing post-operative cases)	152
6	Post-operative refraction/monitoring of refractive outcomes	138
7	Fixed facilities (well-functional base operating theatre)	131
8	Consumables (e.g. viscoelastics, trypan blue)	129
9	Proper case selection	128
10	High volumes of patients (e.g. community referral networks/outreach)	126
11	Refresher training available to surgeons (wet labs)	105
12	Vitrector	95
13	Close follow-up	92
14	Patient education	88
15	Opportunity for anonymous feedback from patients to their surgeon	83
16	External (e.g. MOH) monitoring of cataract surgical outcomes	81
17	Increase number of sub-specialised ophthalmologists (e.g. VR surgeons to deal with complications)	79

## Discussion

Progress towards elimination of cataract blindness as a public health problem in SSA has been slow. The quantity of cataract surgery being performed is still inadequate, and the quality of the surgical service across the continent is believed to be below WHO benchmarks for the visual outcomes of surgery in routine service provision [3, 13].

Evidence from research has led to few candidate interventions to improve the outcomes of surgery. Some interventions, such as routine use of an intra-ocular lens, have been widely taken up [21]. Others, such as surgeons monitoring their outcomes [10, 12, 22–24], the use of intra-cameral cefuroxime as prophylaxis against endophthalmitis [25] or the routine use of biometry [3], have not been so widely adopted in SSA. Biometry utilisation, which is standard in the majority of the world, has not been introduced systematically in SSA by either governments or non-governmental partners, and the contextualised prospective evidence base to drive implementation is lacking.

The underlying assumption of this study is that those involved with service provision have insight into which factors will actually lead to improved surgical quality that patients will regard as better. Regardless of the validity of that assumption, research agendas might be considered to have greater chance of translating into behavioural change if they reflect the priorities perceived by those working within the healthcare systems where development is desired. This Delphi exercise provides a prioritised list of factors, proposed and refined by those active in eye healthcare research and service delivery in SSA, for consideration as the most relevant interventions to be evaluated to improve the objective visual outcomes and patient-reported outcome measures of cataract surgery.

The majority of suggested factors would be targeted at the individual hospital level. Improved surgical training, however, would need engagement of training institutions and universities, and it may be that national Ministry of Health level implementation of the suggested routine monitoring of cataract surgical outcomes helps drive a culture of quality improvement that encourages investigation into other effective interventions.

This is a matter of public health importance. If a cataract surgical rate of 500 is applied to an SSA population of around 800 million, 400,000 operations would be performed annually. However, if 20% of these experience *poor* outcomes (<6/60), then 80,000 people are left worse than 6/60 after cataract surgery, each of whom will likely negatively influence the decision-making of other blind people considering whether or not to present for surgery.

The effort towards improving outcomes is aimed at moving SSA surgical outcomes towards the WHO benchmark of 90% best-corrected good outcome (6/18 or better) and <5% poor outcome (<6/60). If achieved across the continent, this would then reduce the number of people <6/60 post-operatively from 80,000 to 20,000. Before scaling up the volume of surgery being performed, improving the outcomes reported by cataract surgical services is highly desirable.
